# Construct Validity of the Movement Assessment Battery for Children-Second Edition Test in Preschool Children with Respect to Age and Gender

**DOI:** 10.3389/fped.2018.00012

**Published:** 2018-01-30

**Authors:** Jakub Kokštejn, Martin Musálek, James J. Tufano

**Affiliations:** ^1^Faculty of Physical Education and Sport, Charles University, Prague, Czechia

**Keywords:** motor assessments, movement difficulties, confirmatory factor analysis, motor skills, motor development

## Abstract

**Background:**

The Movement Assessment Battery for Children-second edition (MABC-2) Age Band 1 is widely used to identify preschoolers with motor difficulties. Despite unsatisfactory construct validity of the original three-factor model, MABC-2 (manual dexterity, aiming and catching, and balance), previous research has not considered possible age and gender differences throughout the entire preschool period.

**Aim:**

The aim of this study was to verify the construct validity of the MABC-2 Age Band 1 in a population of Czech preschoolers with respect to age and gender.

**Methods:**

Using data from 510 Czech preschoolers (3–6 years; 4.9 ± 1.1 years), confirmatory factor analyses (CFA) were used for each age category and gender.

**Results:**

The goodness-of-fit indices of CFA supported the original three-factor model of the MABC-2 only in 3- and 4-year-old children, and in boys (3–6 years). Low factor loadings and ceiling effects of several test items (Drawing Trail, Walking Heels Raised, and Jumping on Mats) seem to be a probable cause of weak fit indices in 5- and 6-year-old children and in girls (3–6 years).

**Conclusion:**

These results suggest that the MABC-2 can be a valid tool for assessing motor development and identifying motor difficulties among 3- to 4-year olds, and generally fits better for preschool boys in the Czech Republic. However, in 5- to 6-year olds, ceiling effects and a low power of discrimination was found for the Drawing Trail, Walking Heels Raised, and Jumping on Mats tests. Therefore, the three-factor model is not appropriate for all preschoolers, and separate norms should be established for each age and gender.

## Introduction

During childhood, motor development plays a crucial role in the physical, cognitive, and social development of preschool- and school-aged children (1–3). Particularly, the adequate acquisition of fundamental motor skills (FMS) during early childhood has been considered as a crucial step in developing specialized and more complex motor skills later in life (2, 4, 5). Moreover, Rose et al. (6) have argued that a lack of FMS competency may result in frustration and difficulty in learning more specialized skills, thereby reducing the enjoyment of physical activity as well as the likelihood of developing a physically active lifestyle. Therefore, to design effective motor programs or to support the involvement of a child with special needs, it is important to assimilate valid information about the FMS levels of children (7).

To assess motor proficiency and identify impairments in motor coordination in children, standardized motor performance tests are commonly used (3, 8). Of these tests, the second version of the Movement Assessment Battery for Children-second edition (MABC-2) (9) is one of the most commonly used and includes three age bands: age 3–6 (AB1), 7–10 (AB2), and 11–16 (AB3) (3). The MABC-2 test consists of a three-factor model that assesses motor proficiency in three different motor domains: manual dexterity (MD), aiming and catching (AC), and balance (BAL). Based on the total MABC-2 test score, the “traffic light system” identifies a child’s motor competency as fitting into one of three categories: (1) without motor difficulties, (2) at risk of motor difficulties, and (3) severe motor difficulties. Moreover, the final range, with severe motor difficulties, is often associated with the confirmation of a developmental coordination disorder (10).

According to the MABC-2 test manual, the main purposes of the test is to identify motor development problems, evaluate the effectiveness of motor-skill intervention programs, and clinically investigate the motor skills of children (9). Additionally, other child motor development specialists have suggested that the test is suitable for assessing the developmental status of FMS, realizing the achievement of early motor-related milestones, and evaluating specialized movement skills (3, 11). As such, several studies used the MABC-2 to assess and document the levels of FMS competence among normally developing preschool children (12–15). With the MABC-2 being so popular, it is necessary to determine whether the MABC-2 effectively measures separate motor skills in separate domains. If there is a strong relationship between subtest scores, it may be that the separate tests could be measuring similar constructs and essentially counting the score of a shared construct more than once in the total test score (TTS).

Some studies have shown that the MABC-2 test has sufficient content (e.g., MABC-2 tests include different areas of motor skills) and criteria validity (e.g., MABC-2 test scores correlate with motor skills) (16, 17). However, these studies did not enable direct quantitative relationships to be determined between indirectly observed constructs and empirical indicators such as “manual dexterity” and “posting coins,” respectively (16). For assessing the relationship between constructs, or between a construct and a directly measured variable in a definite structure, confirmatory factor analysis (CFA) should be used (17).

In a study conducted by Schulz et al. (18), CFA clearly rejected the MABC-2’s original three-factor model, and the most appropriate model showed a bi-factor structure with one general (motor skill) factor for all variables in the MABC-2 and three separate constructs (MD, AC, and BAL) where correlations between each construct had to be fixed to a value of 0, indicating that they measured different movement properties and were not correlated to each other. These authors, however, did not look at the possible influence of age and gender throughout the entire preschool period, which has recently been shown to affect MABC-2 test scores (13). Similarly, the three-factor model was also rejected by Hua et al. (19) and Psotta and Brom (8) in samples of 1,823 Chinese and 399 Czech preschoolers, respectively. Although Ellinoudis et al. (20) verified the structure of the three-factor model on a sample of Greek preschoolers, they used a relatively small sample size (*n* = 183) that consisted only of 3- to 5-year-old children, excluding 6-year olds, who likely require different testing procedures, scoring procedures, or both (13).

The rather ambiguous results of the aforementioned studies indicate that throughout the preschool period (3–6 years), a wide range of individual differences in motor-skill development is likely present between different ages and genders (13, 21–24). This consideration was supported by Schulz et al. (18), who suggested that future research should examine the structure of factors in the MABC-2 test at different ages. Additionally, the effect of gender could also affect the validity of MABC-2, as research has shown that motor competencies can differ between boys and girls of the same age (13, 14, 25, 26). Specifically, our research group provided evidence that FMS proficiency assessed by the MABC-2 differs between preschool boys and girls. Further, it was found that these differences are not uniform throughout the entire preschool period (3–6 years old) (13). Therefore, we recommended that sex- and age-specific norms should be created for the MABC-2 test. However, as our previous study only assessed the differences between genders and ages, it would be logical that the discriminatory abilities of each test item should also be assessed before new sex- and age-specific norms are developed. By assessing the construct validity of the individual subtests within the MABC-2, it may be possible to make recommendations regarding which test items should remain and which should be adjusted.

Therefore, the aim of this study was to use CFA to verify the construct validity of the MABC-2 test in a Czech population of preschool children with respect to gender and age. We hypothesized that variability in the children’s test performance with respect to age or gender may be the cause of the inconsistent construct validity in the MABC-2 test.

## Materials and Methods

### Participants

A portion of these data (325 children) were previously used to assess whether gender-specific differences in FMS were uniform throughout the entire preschool period (13). As the aims of the present study were starkly different to those of the previous study (13), we also extended our research sample by 185 preschool children to better assess the construct validity of the MABC-2. Therefore, a total of 510 preschool children (4.9 ± 1.1 years; 247 girls and 263 boys) participated in this study. Using gender and age as stratification variables, a stratified sampling method was used to select study participants from 10 randomly selected kindergartens throughout Prague and its surrounding areas. Children who had been previously diagnosed with mental or other serious clinical impairments (*n* = 6) were excluded from the study. In cooperation with the kindergarten’s management, parents were informed on the purpose, benefits, and risks of the study. Those who were interested provided written informed consent for their child’s participation in the study, in accordance with the Declaration of Helsinki. The protocol was approved by the Ethics Committee of the Faculty of Physical Education and Sport at Charles University, Prague. After data collection and analyses, parents received a report with their child’s motor performance results, which also contained information about helpful public service programs for children whose test scores were below the 15th percentile.

### Instrument

The MABC-2 test for age band 1 (3–6 years old) includes eight test items that represent the three motor domains: MD, AC, and BAL (Table 1).

**Table 1:** Movement assessment battery for children-second edition (MABC-2) test for preschool children (age Band 1).

MABC-2 test motor domain	Task
Manual dexterity (fine motor skills)	MD1 posting coins
	MD2 threading beads
	MD3 drawing trail

Aiming and catching (gross motor skills)	AC1 catching beanbag
	AC2 throwing a beanbag onto a mat

Balance	BAL1 one-leg balance
	BAL2 walking heels raised
	BAL3 jumping on mats

According to the MABC-2 manual with norms for Czech preschoolers [Czech version (27)], the raw score achieved in each test item is to be converted into the age-normed standard score. The better a child performs, the higher the standard score is. In the MABC-2 test, the overall level of motor-skill competency is represented by the TTS, which is then calculated as a sum of the standard scores of all eight test items and converted to a standard score equivalent and percentile equivalent. A TTS lesser than or equal to the fifth percentile indicates significant motor difficulties; a TTS between the sixth and 15th percentile indicates a risk of motor difficulties; and a TTS greater than the 15th percentile indicates typical motor coordination development (9).

### Procedure

Children were tested by a team of trained examiners (Master’s degrees in Adapted Physical Education, Special Pedagogy, Physiotherapy, etc.), who underwent the user’s training program that focused on understanding the theoretical issues and practical skills needed for administering and scoring the test. These research assistants performed the same tests for all children, meaning that there were no inter-rater testing procedures. Children were individually tested in their regular educational setting during morning classes, taking about 20–30 min to complete for each child.

### Statistical Analysis

For the purpose of data analysis, we used the standard scores of the MABC-2 test items. To verify the factorial validity of the MABC-2, CFA was used. The Mardia test, Henze–Zirkler’s test, and Royston’s test rejected multivariate normal distribution; therefore, the robust maximum likelihood estimate parameter was used (28, 29). According to the recommendations of McDonald (30) and Maydeu-Olivares and McArdle (31), the following fits were used: (1) model discrepancy: Chi-square (S-Bχ^2^), model significance *p* > 0.05; (2) approximating error: root mean square error of approximation (RMSEA), standardized root mean square residual (SRMR); and (3) incremental fit indices: comparative fit index (CFI), Tucker–Lewis Index (TLI). To determine the quality of a model, we respected the recommendations of McDonald and Marsh (32), Hu and Bentler (33), and Kline (34): RMSEA < 0.06; CFI > 0.95; TLI > 0.95; and SRMR ≤ 0.08.

First, separate CFAs were applied to each age category and then to each gender. Comparisons between model fit between each age category and between genders was done using the Bayesian information criteria (BIC) coefficient: a smaller BIC means a better model fit (29, 35). Differences between two BIC coefficients were evaluated using the approach of Raftery (36) which respects the inner algorithm of the M-plus software, version 6 (29), which was used for data analysis. For revealing possible causes of low fit indices of the model, we checked differences in factor loadings of test items and correlations between factors among each age categories.

Except for the fit indices, the differences between the observed and predicted covariances in residual matrices were investigated. Since the multivariate normality of items was rejected, we analyzed values from the normalized residual matrix (37, 38), as they represent the normalized difference between observed and model predicted model correlation of two variables. This difference is then transformed on scale where values higher than 1.96 are considered to be significant (39, 40). In other words, normalized residuals higher than 1.96 indicate that there is a large unexplained portion of a relationship between the empirical and predicted correlation of two variables. Additionally, the frequencies of a child achieving the maximum score in each test (i.e., ceiling effects) were also evaluated.

## Results

**Figure 1:**
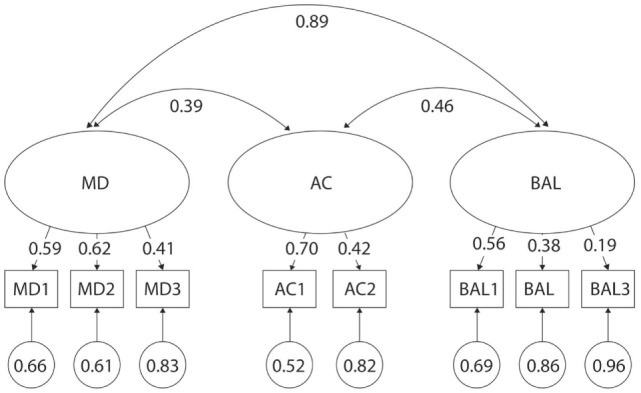
Original three-factor model of the movement assessment battery for children-second edition-2 test for 3- to 6-year-old children. MD1–BAL3: test items; latent factors: MD, manual dexterity; AC, aiming and catching; BAL, balance.

For the sake of simplicity, only data that do not support the three-factor model are presented in the text and it can be assumed that data that is not reported in the text support the three-factor model. When assessing children of both genders and all ages together, the original three-factor was rejected according to the significant chi-square value (*p* < 0.01) and the poor fit indices (CFI < 0.95, TLI < 0.95) (Table 2). Subsequent analysis of factor loading differences revealed poor discriminatory properties (Figure 1). For example, although high factor loading values (λ) are desired, they were as low as 0.19 for BAL3 Jumping on Mats. Moreover, a very high correlation was found between MD and BAL (*r* = 0.89), suggesting that there is poor discrimination between these two behaviorally different constructs.

**Table 2:** Fit indices of the original three-factor model of movement assessment battery for children-second edition for all children; girls of all ages and boys of all ages; and boys and girls combined for separate age categories.

Group	*N*	S-Bχ^2^	*P*	DF	BIC	RMSEA	RMSEA 90% CI	SRMR	CFI	TLI
All children	510	41.44	0.008	17	19,275.71	0.053	0.033–0.074	0.040	0.92	0.87
Girls: all ages	246	48.01	<0.000	17	9,968.60	0.086	0.058–0.115	0.069	0.79	0.66
Boys: all ages	263	20.61	0.240	17	9,276.97	0.028	0.000–0.066	0.038	0.98	0.96
3-year olds	121	17.82	0.400	17	4,932.55	0.020	0.000–0.086	0.050	0.99	0.98
4-year olds	143	19.14	0.320	17	5,149.36	0.030	0.000–0.084	0.039	0.98	0.97
5-year olds	125	27.85	0.050	17	5,578.96	0.070	0.009–0.118	0.074	0.85	0.75
6-year olds	121	20.75	0.240	17	5,477.35	0.043	0.001–0.096	0.058	0.86	0.76
3- to 4-year-old boys	142	24.50	0.106	17	5,692.82	0.056	0.000–0.101	0.045	0.94	0.89
3- to 4- year-old girls	122	31.37	0.018	17	5,826.58	0.083	0.034–0.128	0.059	0.88	0.80
5- to 6-year-old boys	121	18.36	0.364	17	4,231.36	0.026	0.000–0.088	0.053	0.98	0.97
5- to 6-year-old girls	125	Heywood case correlation between BAL1 and factor balance greater than 1								

*S-Bχ^2^, chi-squared; DF, degrees of freedom; BIC, Bayesian information criteria; RMSEA, root mean square error of approximation; CI, confidence interval; SRMR, standardized root mean square residual; CFI, comparative fit index; and TLI, Tucker–Lewis index*.

*Greater CFI, TLI, and p-values are desirable, indicating a “better” model fit; whereas lower BIC, RMSEA, and SRMR are desirable, indicating a “better” model fit*.

**Figure 2:**
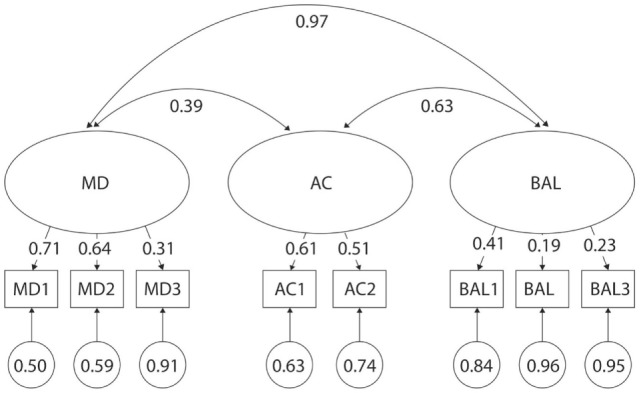
Original three-factor model of the movement assessment battery for children-second edition test for 3- to 6-year-old girls. MD1–BAL3: test items; latent factors: MD, manual dexterity; AC, aiming and catching; BAL, balance.

**Figure 3:**
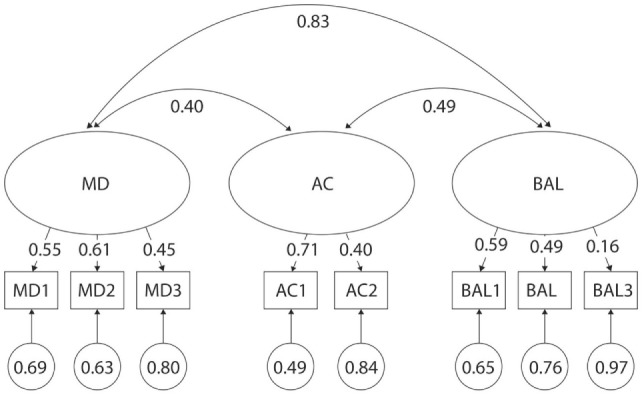
Original three-factor model of the movement assessment battery for children-second edition test for 3- to 6-year-old boys. MD1–BAL3: test items; latent factors: MD, manual dexterity; AC, aiming and catching; BAL, balance.

When combining children of all ages together but assessing each gender independently, the three-factor model of the MABC-2 test did not fit in girls (S-Bχ^2^ = 48.01. *p* < 0.01) with the lowest fit indices of all analyses within this study (TLI = 0.66, CFI = 0.79) (Table 2). Subsequent analysis of factor loading differences revealed the lowest discriminatory properties with λ values as low as 0.31 in girls for MD3—Drawing Trail, 0.19 in girls for BAL2—Walking Heels Raised, and 0.23 and 0.16 in both girls and boys, respectively, for BAL3—Jumping on Mats. Additionally, the correlation was too high in girls between factors MD and BAL (*r* = 0.97 and *r* = 0.83) (Figures 2 and 3). Normalized residual matrices were not satisfactory (>1.96) in girls between MD3—Drawing Trail and BAL1—One-Leg BAL = 2.373; AC2—Throwing a Beanbag onto a Mat and BAL2—Walking Heels Raised = 2.172; and AC2—Throwing a Beanbag onto a Mat and BAL3—Jumping on Mats = 1.973.

When combining both genders together but assessing each age category independently, CFA showed that the original three-factor model of MABC-2 did not fit equally across all ages (Table 2). Although the RMSEA, SRMR, and *p*-values generally suggested that empirical covariances of MABC-2 items agreed with the predicted model covariances in all age categories, different BIC index values and poor CFI and TLI scores revealed significant variability in model fits between age categories.

In the next step of our analysis, we divided children according to age and gender, focusing on age and gender interactions. Subsequently, four CFAs (3- to 4-year-old boys, 3- to 4-year-old girls, 5- to 6-year-old boys, and 5- to 6-year-old girls) revealed that for both age groups of boys, the original three-factor model fits well. On the other hand, the fit was not as good in girls of all age groups, especially in 5- to 6-year-old girls where a Heywood case was detected. A Heywood case represents negative variance which indicates that there can be any combination of problems within the model such as too little variance of a directly measured item explained in construct, an item discrimination that is either too high or too low, a singularity in the matrix, an unusual random sample, or other causes. In our study, BAL1 showed the greatest variance compared to BAL2 and BAL3. It seemed that in the model, the majority of variance of construct was, therefore, explained by BAL1 and other items did not contribute significantly. As a result of the analysis for 5- to 6-year-old girls, it is not possible to compose a construct by one significant item. In other words, the results in 5- to 6-year-old girls showed too little variance to be explained in the construct, thus forcing structural error variance to be negative.

**Figure 4:**
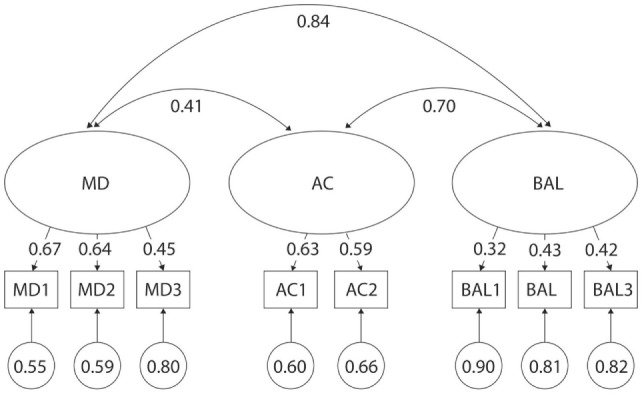
Original three-factor model of the movement assessment battery for children-second edition test for 3-year-old children. MD1–BAL3: test items; latent factors: MD, manual dexterity; AC, aiming and catching; BAL, balance.

**Figure 5:**
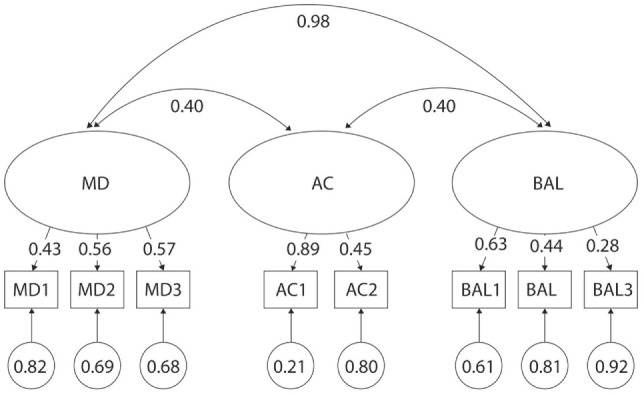
Original three-factor model of the movement assessment battery for children-second edition test for 4-year-old children. MD1–BAL3: test items; latent factors: MD, manual dexterity; AC, –aiming and catching; BAL, balance.

**Figure 6:**
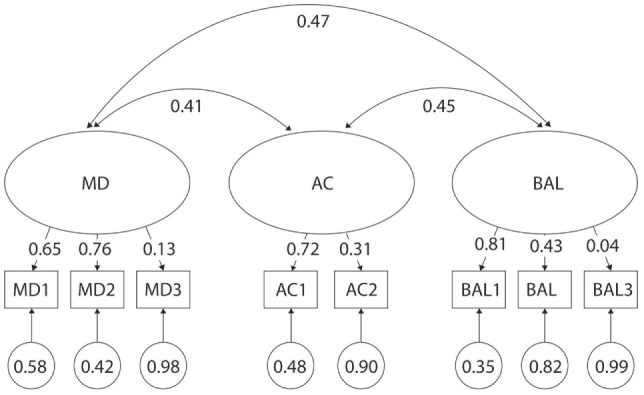
Original three-factor model of the movement assessment battery for children-second edition test for 5-year-old children. MD1–BAL3: test items; latent factors: MD, manual dexterity; AC, aiming and catching; BAL, balance.

**Figure 7:**
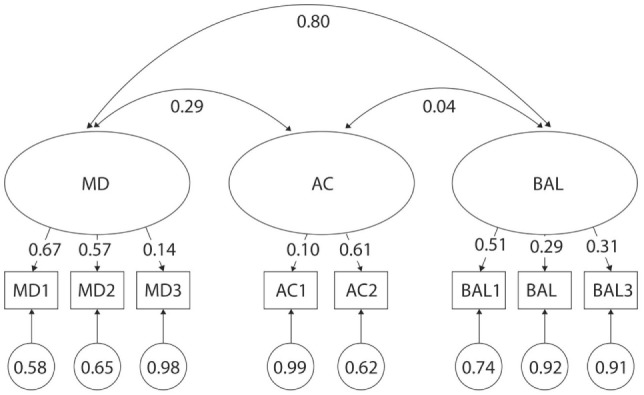
Original three-factor model of the movement assessment battery for children-second edition test for 6-year-old children. MD1–BAL3: test items; latent factors: MD, manual dexterity; AC, aiming and catching; BAL, balance.

Several test items from the MABC-2 had poor discriminatory properties across age categories (Figures 4–7). For example, λ values were as low as λ = 0.13 and 0.14 in 5- and 6-year olds, respectively for MD3—Drawing; λ = 0.10 in 6-year olds for AC1—Catching Beanbag; λ = 0.31 in 5-year olds for AC2—Throwing a Beanbag onto a Mat; λ = 0.32 in 3-year olds for BAL1—One-Leg BAL; and λ = 0.04, 0.28, and 0.31 in 5-, 4-, and 6-year olds, respectively for BAL3—Jumping on Mats. Additionally, correlations were too high between certain factors across age categories (Figures 4–7). For example, *r* values were as high as 0.80, 0.84, and 0.98 for MD and BAL in 6-, 3-, and 4-year olds, respectively. Normalized residual matrices were satisfactory (<1.96) for 3- and 4-year olds, but not 5- and 6-year olds (>1.96). In 5-year olds, the normalized residual was 3.166 for MD3—Drawing Trail and AC2—Throwing a Beanbag onto a Mat; and 2.690 for MD3—Drawing Trail and BAL2—Walking Heels Raised. In 6-year olds, the normalized residual was 2.134 for AC2—Throwing a Beanbag onto a Mat and BAL2—Walking Heels Raised. Moreover, a very strong ceiling effect (high percentage of children who reached the maximal score) was found in BAL2—Walking Heels Raised (78 and 85% in 5- and 6-year olds, respectively), BAL3—Jumping on Mats (94 and 95% in 5- and 6-year olds, respectively), and MD3—Drawing Trail (70% in 6-year olds).

## Discussion

Based on the results of previous studies (8, 18, 19), we hypothesized that variability in children’s motor performance in relation to age or gender may be the underlying origin of inconsistent psychometric properties in the MABC-2 test. To our knowledge, this is the first study to verify the, apparent lack of, factorial validity of the original three-factor model of the MABC-2 motor test for each age category separately during the entire preschool period while also accounting for gender. As hypothesized, the three-factor model’s goodness-of-fit indices were not satisfactory when grouping all (*n* = 510) preschool children together (RMSEA = 0.053; SRMR = 0.040; CFI = 0.92; TLI = 0.987). Specifically, the model did not fit when girls of all ages were grouped together, nor did it fit for 5-year olds or 6-year olds when boys and girls of the same age were grouped together. Moreover, the most serious problems were found in 5- to 6-year-old girls where the model showed a Heywood case, identifying problems specifically in the BAL construct. However, the present model did fit for 3-year olds of both genders, 4-year olds of both genders, and boys of all ages. All things considered, the findings of the present study suggest that the current three-factor model should not be applied to preschoolers of all ages and both genders, and that age- and gender-specific testing and scoring procedures should be developed.

Other researchers have also noted that the three-factor model may not accurately explain the empirical data that the MABC-2 purports to unveil (8, 18, 19). Particularly low factor loadings and a large number of standardized residuals were the main causes of the unsatisfactory fit of the original model of the MABC-2 in these studies. To obtain a satisfactory fit, the authors subsequently made additional statistical adjustments (excluding the weak test items, adding the correlated measurement errors, double factor loading of some test items, or creating general motor factor). Although the authors tried to defend their modification of the original model of MABC-2 test, such adjustments often decrease the theoretical nature of the model’s constructs. Despite modifying these variables to achieve good fit indices of the model, these authors did not consider the possible effect of age and gender in the entire population of preschool children. Only Psotta and Brom (8) divided the sample into younger (3–4 years old) and older (5–6 years old) preschoolers while attempting to verify the construct validity of the MABC-2, but the authors did not report a satisfactory fit of the original three-factor model for either of the two age groups.

With respect to age, the results of CFA in the present study revealed substantial variability between different age categories. The findings clearly suggest that the three-factor model appears to sufficiently fit for 3- and 4-year-old Czech children independently, but not for 5- and 6-year olds. In contrast to a general motor factor as a possible indicator of children’s overall motor competence (18), our results support the validity of the original three-factor model MABC-2 which is able to distinguish motor performance in different motor domains (fine motor skills, gross motor skills, and BAL) only in 3- and 4-year-old children.

We found substantial variability of factor loadings in MD3 (0.13–0.57), AC1 (0.10–0.89), BAL1 (0.32–0.81), BAL3 (0.04–0.42) between age categories. These findings showed that the aforementioned test items discriminate the level of three motor domains in significantly different ways across the entire preschool period. Moreover, we also revealed very low factor loadings of MD3, BAL2, and BAL3. These findings support the results of previous research where MD3 and BAL2 (19), MD3 and BAL3 (18), and BAL2 and BAL3 (8) were also identified as problematic due to their poor factor validity. Thus, the results suggest that these test items probably do not sufficiently represent their corresponding motor domains.

Although determining the construct validity was the primary aim of this study, our results identified a somewhat problematic phenomenon: many children achieved the highest possible score in some tests, indicating that a ceiling effect was present. The purpose of the MABC-2 test is to identify children who may be at risk of developing motor difficulties. However, if scoring procedures are too lenient or the test is too easy, the scores may artificially inflate a child’s overall FMS performance, masking a possible motor impairment. In our study, a large percentage of children achieved the highest possible score in BAL2 (78 and 85% in 5- and 6-year olds, respectively), BAL3 (94 and 96% in 5- and 6-year olds, respectively) and MD3 (70% in 6-year olds), similar to Psotta and Brom (8). Thus, the tests are either likely too easy for 5- and 6-year olds, or the scoring criteria are too lenient, both indicating that the tests may not be able to discriminate between children who lack motor deficiencies, those at risk of deficiency, or those who have sever motor impairments. To further investigate the possible problematic nature of the testing and scoring procedures for these tests, we determined the discrimination function of MD3, BAL2, and BAL3 in relation to a TTS. An agreement between poor performance (≤16th percentile) in MD3, BAL2, and BAL3 and poor TTS (≤16th percentile) was 65% in 3-year olds, 60% in 4-year olds, 44% in 5-year olds, and 47% in 6-year olds. Thus, a strong ceiling effect and weak ability of discrimination for MD3, BAL2, and BAL3 appear as possible causes of the low discriminatory ability in dynamic BAL and MD in 5- and 6-year-old preschool children.

The original three-factor model showed good fit indices in boys (3–6 years), but the goodness-of-fit indices for girls were not satisfactory. Poor factor loadings of MD3 and BAL2 in girls and BAL3 in both genders suggests that these manifest variables likely measure different latent variables. This suggestion is supported by a very high correlation between the MD and BAL MABC-2 subtests (*r* = 0.97 in girls and *r* = 0.83 in boys). However, this assumption is hypothetical and cannot be determined using the data at hand. On the contrary, low factor loadings (poor discrimination property) of MD3 and BAL3 could be due to the presence of ceiling effects, indicating that the test requirements are too easy, or the scoring criteria are too lenient. Double factor loading, when manifest variables are significantly related to two latent factors, were also found in studies of Schulz et al. (18) and Psotta and Brom (8). Another possible explanation for the differences in the model fit indices for both genders in our study could be different rates in motor development between boys and girls. Recently, studies have shown that preschoolers develop FMS at different rates (13, 25, 26) and that these differences are not uniform through throughout the entire preschool period (13). As a result, consideration for separated norms for boys and girls had already been suggested (13, 14), and are again affirmed here.

Ambiguous results about the quality of the original three-factor model of MABC-2 were found in our study with respect to age and gender during entire preschool period. Particularly, low factor loadings and ceiling effects of several test items seem to be possible problems of the unsatisfactory construct validity of MABC-2 in 5- and 6-year-old children, and especially in girls 3–6 years old. The data from the present study confirm the suggestions set forth by Kokštejn et al. (13) and Livesey et al. (14) that gender-specific normative values should be determined so that the MABC-2 can effectively identify children with motor difficulties, ultimately resulting in more appropriate motor intervention programs for preschool children.

## Author Contributions

All authors contributed equally to this article.

## Conflict of Interest Statement

The authors declare that the research was conducted in the absence of any commercial or financial relationships that could be construed as a potential conflict of interest.

## References

[1] Gabbard CP Lifelong Motor Development: Pearson New International Edition. 6th ed Philadelphia: Pearson Higher Education (2013).

[2] Gallahue DL Ozmun JC Goodway JD Understanding Motor Development: Infants, Children, Adolescents, Adults. 7th ed New York: McGraw-Hill (2011).

[3] Cools W De Martelaer K Samaey C Andries C. Movement skill assessment of typically developing preschool children: a review of seven movement skill assessment tools. J Sports Sci Med (2009) 8(2):154–68. 24149522 PMC3761481

[4] Stodden D Goodway J Langendorfer S Roberton M Rudisill M Garcia C A developmental perspective on the role of motor skill competence in physical activity: an emergent relationship. Quest (2008) 60(2):290–306. 10.1080/00336297.2008.10483582

[5] Clark JE Metcalfe JS The mountain of motor development: a metaphor. Motor Dev Res Rev. Reston, VA: NASPE (2002) 2:163–90.

[6] Rose B Larkin D Berger BG The importance of motor coordination for children’s motivational orientations in sport 1. Adapt Phys Activ Q (1998) 15(4):316–27. 10.1123/apaq.15.4.316

[7] Hands BP How can we best measure fundamental movement skills? Health Sciences Conference Papers Launceston Tas (2002). 5 p.

[8] Psotta R Brom O. Factorial structure of the movement assessment battery for children test-second edition in preschool children. Percept Mot Skills (2016) 123(3):702–16. 10.1177/0031512516666072 27590494

[9] Henderson SE Sugden DA Barnett AL Movement Assessment Battery for Children-2 (MABC-2): Examiner’s Manual. London: Pearson Assessment (2007).

[10] American Psychiatric Association. Diagnostic and Statistical Manual of Mental Disorders. 5th ed Arlington: American Psychiatric Publishing (2013).

[11] Burton AW Miller DE Movement Skill Assessment. Champaign: Human Kinetics (1998).

[12] Fisher A Reilly JJ Kelly LA Montgomery C Williamson A Paton JY Fundamental movement skills and habitual physical activity in young children. Med Sci Sports Exerc (2005) 37(4):684–8. 10.1249/01.MSS.0000159138.48107.7D 15809570

[13] Kokštejn J Musálek M Tufano JJ. Are sex differences in fundamental motor skills uniform throughout the entire preschool period? PLoS One (2017) 12(4):e0176556. 10.1371/journal.pone.0176556 28448557 PMC5407840

[14] Livesey D Coleman R Piek J. Performance on the movement assessment battery for children by Australian 3- to 5-year-old children. Child Care Health Dev (2007) 33(6):713–9. 10.1111/j.1365-2214.2007.00733.x 17944780

[15] Ziviani J Poulsen A Hansen C Movement skills proficiency and physical activity: a case for engaging and coaching for health (EACH)–child. Aus Occup Ther J (2009) 56(4):259–65. 10.1111/j.1440-1630.2008.00758.x 20854526

[16] Wagner MO Kastner J Petermann F Bös K. Factorial validity of the movement assessment battery for children-2 (age band 2). Res Dev Disabil (2011) 32(2):674–80. 10.1016/j.ridd.2010.11.016 21146955

[17] Brown T Lalor A The movement assessment battery for children—second edition (MABC-2): a review and critique. Phys Occup Ther Ped (2009) 29(1):86–103. 10.1080/01942630802574908 19197761

[18] Schulz J Henderson SE Sugden DA Barnett AL. Structural validity of the movement ABC-2 test: factor structure comparisons across three age groups. Res Dev Disabil (2011) 32(4):1361–9. 10.1016/j.ridd.2011.01.032 21330102

[19] Hua J Gu G Meng W Wu Z. Age band 1 of the movement assessment battery for children-second edition: exploring its usefulness in mainland China. Res Dev Disabil (2013) 34(2):801–8. 10.1016/j.ridd.2012.10.012 23220119

[20] Ellinoudis T Evaggelinou C Kourtessis T Konstantinidou Z Venetsanou F Kambas A Reliability and validity of age band 1 of the movement assessment battery for children-second edition. Res Dev Disabil (2011) 32(3):1046–51. 10.1016/j.ridd.2011.01.035 21333488

[21] Gabbard CP Lifelong Motor Development. 7th ed Philadelphia: Pearson Higher Education (2011).

[22] Kambas A Venetsanou F Giannakidou D Fatouros IG Avloniti A Chatzinikolaou A The motor-proficiency-test for children between 4 and 6 years of age (MOT 4–6): an investigation of its suitability in Greece. Res Dev Disabil (2012) 33(5):1626–32. 10.1016/j.ridd.2012.04.002 22543059

[23] Van Waelvelde H Peersman W Lenoir M Smits Engelsman B Henderson S. The movement assessment battery for children: similarities and differences between 4- and 5-year-old children from Flanders and the United States. Pediatr Phys Ther (2008) 20(1):30–8. 10.1097/PEP.0b013e31815ee2b2 18300931

[24] Gidley Larson JC Mostofsky SH Goldberg MC Cutting LE Denckla MB Mahone EM. Effects of gender and age on motor exam in typically developing children. Dev Neurol (2007) 32(1):543–62. 10.1080/87565640701361013 17650993 PMC2099302

[25] Hardy L King L Farrell L Macniven R Howlett S Fundamental movement skills among Australian preschool children. J Sci Med Sport (2009) 13(5):503–8. 10.1016/j.jsams.2009.05.010 19850520

[26] Foulkes J Knowles Z Fairclough S Stratton G O’dwyer M Ridgers N Fundamental movement skills of preschool children in northwest England. Percept Mot Skills (2015) 121:260–83. 10.2466/10.25.PMS.121c14x0 26270852

[27] Psotta R MABC-2: Test motoriky pro děti (1. českévydání) [The Movement Assessment Battery for Children-2 Test (1st Czech edition)]. Praha, Czech Republic: Hogrefe-Testcentrum (2014).

[28] Ferron JM Hess MR Estimation in SEM: a concrete example. J Behav Exp x (2007) 32(1):110–20. 10.3102/1076998606298025

[29] Muthén LK Muthén BO Mplus: Statistical Analysis with Latent Variables: User’s Guide. Los Angeles: Muthén&Muthén (2010).

[30] McDonald RP Test Theory: A Unified Treatment. Mahwah, NJ: L. Erlbaum Associates (1999).

[31] Maydeu-Olivares A McArdle JJ Contemporary Psychometrics. Mahwah NJ: Lawrence Erlbaum (2005).

[32] McDonald RP Marsh HW Choosing a multivarite model: noncentrality and goodness of fit. Psychol Bull (1990) 107(2):247–55. 10.1037/0033-2909.107.2.247

[33] Hu L Bentler PM Fit indices in covariance structure modeling: sensitivity to underparametrized model misspecification. Psychol Methods (1998) 3(4):424–53. 10.1037//1082-989X.3.4.424

[34] Kline RB Principles and Practice of Structural Equation. 3rd ed New York: Guildford Press (2011).

[35] Findley DF Counter examples to parsimony and BIC. Ann Inst StatMath (1991) 43:505–14. 10.1007/BF00053369

[36] Raftery AE Bayesian model selection in social research. Sociol Methodol (1995) 25:111–63. 10.2307/271063

[37] Bollen KA Structural Equations with Latent Variables. New York: Wiley Interscience (1989).

[38] Hausman JA Specification tests in econometrics. Econometrica (1978) 46(6):1251–71. 10.2307/1913827

[39] Jöreskog KG Sörbom D LISREL 8 User’s Reference Guide. Chicago, IL: Scientific Software International (1993).

[40] Brown TA Confirmatory Factor Analysis for Applied Research. New York: Guilford Press (2006).

